# Characterization of the neural circuitry of the auditory thalamic reticular nucleus and its potential role in salicylate-induced tinnitus

**DOI:** 10.3389/fnins.2024.1368816

**Published:** 2024-04-02

**Authors:** Qian Dai, Tong Qu, Guoming Shen, Haitao Wang

**Affiliations:** School of Integrated Chinese and Western Medicine, Anhui University of Chinese Medicine, Hefei, China

**Keywords:** thalamic reticular nucleus, viral tracing, tinnitus, auditory cortex, salicylate

## Abstract

**Introduction:**

Subjective tinnitus, the perception of sound without an external acoustic source, is often subsequent to noise-induced hearing loss or ototoxic medications. The condition is believed to result from neuroplastic alterations in the auditory centers, characterized by heightened spontaneous neural activities and increased synchrony due to an imbalance between excitation and inhibition. However, the role of the thalamic reticular nucleus (TRN), a structure composed exclusively of GABAergic neurons involved in thalamocortical oscillations, in the pathogenesis of tinnitus remains largely unexplored.

**Methods:**

We induced tinnitus in mice using sodium salicylate and assessed tinnitus-like behaviors using the Gap Pre-Pulse Inhibition of the Acoustic Startle (GPIAS) paradigm. We utilized combined viral tracing techniques to identify the neural circuitry involved and employed immunofluorescence and confocal imaging to determine cell types and activated neurons.

**Results:**

Salicylate-treated mice exhibited tinnitus-like behaviors. Our tracing clearly delineated the inputs and outputs of the auditory-specific TRN. We discovered that chemogenetic activation of the auditory TRN significantly reduced the salicylate-evoked rise in c-Fos expression in the auditory cortex.

**Discussion:**

This finding posits the TRN as a potential modulatory target for tinnitus treatment. Furthermore, the mapped sensory inputs to the auditory TRN suggest possibilities for employing optogenetic or sensory stimulations to manipulate thalamocortical activities. The precise mapping of the auditory TRN-mediated neural pathways offers a promising avenue for designing targeted interventions to alleviate tinnitus symptoms.

## Introduction

Subjective tinnitus, the perception of non-existent sounds, burdens an estimated 14% of adults globally, with potentially severe consequences including anxiety, depression, and, in extreme cases, suicide (Rauschecker et al., 2010; Roberts et al., 2010; Leaver et al., 2011; Elgoyhen et al., 2015; Henton and Tzounopoulos, 2021; Peng et al., 2023). Despite its prevalence, there are currently no effective pharmaceutical treatments available for tinnitus (Henton and Tzounopoulos, 2021). Noise-induced trauma and ototoxic medications are the leading culprits (Eggermont and Roberts, 2004; Rauschecker et al., 2010; Roberts et al., 2010). Salicylate, a common anti-inflammatory and analgesic agent, is routinely used to elicit tinnitus in animal models (Guitton et al., 2003; Puel and Guitton, 2007; Ruel et al., 2008; Su et al., 2012; Yi et al., 2016). In addition to the commonly used rodent animal models, salicylate-induced tinnitus studies have also been conducted in non-rodent animals. For instance, in monkeys administered salicylate orally, tinnitus-like behaviors and altered neural activity in the auditory cortex were observed, consistent with findings in rodent models (Rogenmoser et al., 2022). Similarly, cats receiving intraperitoneal salicylate injection exhibited increased spontaneous neural activity in the auditory system (Ochi and Eggermont, 1996; Eggermont and Kenmochi, 1998; Eggermont, 2008). These studies in rodent and non-rodent animals support the validity of salicylate-induced tinnitus models and provide valuable insights into the underlying mechanisms of tinnitus.

Research suggests that central neural plasticity, characterized by increased spontaneous neural firing and enhanced synchrony, underpins tinnitus pathology—a result of diminished central inhibition (Eggermont and Roberts, 2004; Llinas et al., 2005; Weisz et al., 2007; Roberts et al., 2010; Stolzberg et al., 2011; Llano et al., 2012; Vianney-Rodrigues et al., 2019). Within the auditory cortex, inhibitory GABAergic interneurons serve to dampen neural excitability (Wang et al., 2006; Su et al., 2009; Studer and Barkat, 2022). Conversely, in the thalamus, particularly in the medial geniculate body (MGB), GABAergic neurons are sparse (Winer and Wenstrup, 1994; Winer et al., 1999; Llinas et al., 2005). Instead, the thalamic reticular nucleus (TRN) provides potent inhibitory inputs to the thalamus, modulating its output through reciprocal connections that dictate thalamocortical rhythmicity (Steriade et al., 1993; Contreras et al., 1996; Steriade, 2005; Halassa et al., 2014). In a unique anatomical position, the TRN is referred to as the “gatekeeper” of thalamocortical information flow, influencing processes like change detection, attention, and consciousness (Steriade, 1996; Steriade, 2005; Yu et al., 2009a; Li et al., 2020). It consists of distinct sectors serving limbic and various sensory modalities—visual, auditory, and somatosensory—as evidenced by anatomical and physiological data (Yu et al., 2009a, 2011; Dong et al., 2019; Martinez-Garcia et al., 2020; Wang et al., 2023). The auditory sector of the TRN (aTRN), in particular, receives and processes ascending auditory signals, exerts inhibitory control over the MGB, and shows more stimulus-specific adaptation, emphasizing its role in auditory attention (Yu et al., 2009a,b; Antunes and Malmierca, 2014). Inputs from visual systems and limbic regions, such as the visual cortex and the amygdala, suggest the TRN’s role in multi-sensory integration and affective aspects of auditory processing (Yu et al., 2009a; Aizenberg et al., 2019).

Given the critical role of inhibition in generating tinnitus and the inhibition mediated by TRN (Steriade, 2005; Llano et al., 2012; Galazyuk et al., 2019), boosting TRN-mediated inhibition may relieve tinnitus-related hyperactivity within the central auditory system (Richardson et al., 2012). Traditional tracing methods, such as horseradish peroxidase, lack cellular specificity, and electrophysiological recordings fall short of accurately demarcating the boundaries of aTRN (Yu et al., 2009a; Kimura et al., 2012). However, due to their efficiency and trans-synaptic properties, viral tracing techniques have revolutionized the mapping of neural circuits, particularly when combined with transgenic mice expressing Cre recombinase, which allows for cell type-specific targeting (Xu et al., 2020; Liu et al., 2022). Given the diversity of inputs that converge on the TRN, we endeavored to delineate the specific input–output relationships of the aTRN.

In the current study, we employed a sodium salicylate-induced mouse model of tinnitus to probe the role of the TRN. We utilized anterograde viral tracing to localize the aTRN, and complementary retrograde tracing to elucidate the broader neural networks that feed into and emanate from it. Chemogenetic activation of the aTRN was utilized to assess the potential ameliorative effects on tinnitus-related neural hyperactivity. A comprehensive understanding of the aTRN’s neural circuitry may pave the way for novel therapeutic strategies that enhance central inhibition and benefit those suffering from tinnitus.

## Materials and methods

### Animals

Male C57BL/6 J, CaMKII-Cre, and *Gad2-Cre* mice, aged 8–10 weeks and sourced from Jackson Laboratory or Charles River, were housed in stable environmental conditions with a constant ambient temperature of 23–25°C, 50% humidity, and maintained on a 12:12 light–dark cycle (lights on 07:00–19:00). They had *ad libitum* access to food and water and were grouped five per cage. All procedures were in accordance with ethical standards and approved by the Animal Care Committee of Anhui University of Chinese Medicine. Mice underwent auditory brainstem response (ABR) testing to confirm the absence of hearing deficits before inclusion in behavioral experiments.

### Auditory brainstem response recording

ABR recordings were conducted to assess the hearing ability of the mice included in the study. The Tucker-Davis Technologies System 3 hardware, including Medusa Pre-Amps, RZ6 Multi I/O Processor, and MF1 multifield magnetic speaker, along with BioSigRZ 5.7 software, were utilized for sound delivery and ABR recordings. Acoustic stimuli were delivered through an open-field magnetic speaker (MF1; Tucker-Davis Technologies), using click-pips as auditory stimuli. For the collection of sound-evoked bioelectrical potentials, three needle electrodes were inserted subcutaneously in the contralateral ear (reference), the vertex of the head (ground), and the test ear (active) of isoflurane-anesthetized mice. Hearing thresholds were determined as the minimum sound intensity required to evoke an ABR waveform in which wave I was visually identifiable.

### Stereotactic surgery and virus injection

As described before (Wang et al., 2019; Zhou et al., 2022), mice were anesthetized with an intraperitoneal injection of pentobarbital (20 mg/kg) and secured in a stereotactic frame (RWD Life Science) with body temperatures maintained at 36°C using a heating pad. Surgically, after a craniotomy based on coordinates from the mouse brain atlas, virus injections were performed by delivering 100–250 nL viral solutions into targeted regions at a rate of 30 nL/min using a glass micropipette attached to a Hamilton syringe by a microinjection syringe pump (UMP3T-1; World Precision Instruments). Following injection, the pipette remained in place for 5 min to prevent backflow, and ocular moisture was preserved with ointment. Target coordinates based on the mouse brain atlas (Paxinos and Keith, 2001) were: medial geniculate body (MGB) (−3.35 mm AP, 2.20 mm ML, 3.26 mm DV) and auditory thalamic reticular nucleus (TRN) (−1.27 mm AP, 2.47 mm ML, 3.30 mm DV). Anterograde trans-synaptic tracing leveraged AAV-hSyn-DIO-mCherry (AAV2/9, 2.12 × 10^12^ vg ml^−1^, 150 nL) and AAV-hSyn-Cre-GFP (AAV2/9, 2.12 × 10^12^ vg ml^−1^, 150 nL) viruses. For retrograde trans-synaptic tracing, the helper of AAV-EF1a-DIO-GFP-T2A-TVA (AAV2/9, 2 × 10^12^ vg ml − 1, 100 nL) and AAV-EF1a-DIO-RVG (AAV2/9, 2 × 10^12^ vg ml − 1,100 nL) was injected to enable RV infection by expressing TVA and restoring RV trans-synaptic capability, respectively; 14 days later the virus of RV-EnvA-ΔG-dsRed (2 × 10^8^ IFU/mL, 150 nL) was delivered at the same site. Chemogenetic activation used rAAV-Ef1α-DIO-hM3D(Gq)-mCherry-WPRE-pA (AAV-DIO-hM3Dq-mCherry, AAV2/9, 5.00 × 10^12^ vg ml^−1^, 150 nL) viruses in the Gad2-Cre mice. All viruses were sourced from BrainVTA (Wuhan, China).

### Behavioral testing for tinnitus

C57 mice were divided into saline and salicylate groups for tinnitus behavior analysis via the gap-prepulse inhibition of the acoustic startle (GPIAS). The experimental program was designed in RPvdsEX of TDT system 3, and run with OpenEX software. Mice were placed in a self-made tube (32 mm in diameter and 10 cm in length). The startle reflex was detected by a pressure sensor (Honeywell, FSG15N1A), amplified by an RZ6 processor, and then collected on a computer for off-line analysis (TDT, United States). Behavioral tests were performed after the mice were fully acclimated to the experimental environment (10 min/day, about 3 days). The sound pressure level (SPL) was calibrated using a condenser microphone (PCB Precision Condenser, PCB Piezotronics, NY). The whole experiment can be divided into 4 blocks: Block 1 was the adaptation period of playing background white noise (65 dB SPL) (5 min). Block 2 consisted of 10 trials with a sound intensity of 115 dB SPL and a 20 ms wide-band noise pulse occurring randomly between the 4th and 8th seconds of each trial. The startled stimulus was delivered by a speaker placed 13 cm on the side of the animal (Model CP-75A, Chuangmu Sound). Block 3 consisted of a GPIAS test protocol with a silence gap on a continuous narrow-band noise as a suppression-producing pre-stimulus, and narrow-band background noise with different center frequencies (9, 12, 16, 24, 28 kHz; Bandwidth 1 kHz) at 65 dB SPL was played through the MF1 speaker (TDT). Each frequency background noise was played by mixing 10 trials (gaps) with a silent gap (50 ms) and 10 trials (no gaps) with no silent gaps in random order. Each trial lasted 10 s, and the silence gap appeared 50 ms before the startle stimulus. Block 4, like Block 2, detected whether animals have adapted to the startle reflex. The Startle Ratio is Startle Amplitude (gap)/Startle Amplitude (no gap). The pre-pulse inhibition (PPI) testing apparatus was the same as that of GPIAS testing. The difference was that background sound was absent, and narrow-band noise centered at frequencies 9, 12, 16, 24, and 28 kHz (Bandwidth 1 kHz) was presented 50 ms before the startle noise. The data analysis was accomplished using the custom-written script program in Matlab 2015b.

### Chemogenetics and chemical administration

For chemogenetic manipulations, mice were anesthetized with isoflurane and intraperitoneally administered with either clozapine N-oxide (CNO), the ligand for hM3Dq receptors, at a dosage of 5 mg/kg (Sigma) or a comparable volume of saline. CNO injection was done 30 min before the intraperitoneal injection of sodium salicylate (250 mg/kg) to induce tinnitus. An hour post-salicylate administration, mice were scarified for subsequent immunohistochemical staining.

### Immunohistochemistry and imaging

After deep anesthesia with pentobarbital sodium (20 mg/kg, i.p), mice underwent transcardial perfusion with saline followed by 4% paraformaldehyde (PFA). Brains were extracted, post-fixed in 4% PFA overnight at 4°C, and cryoprotected in 30% sucrose solution. Coronal brain sections (40 μm) were sliced on a cryostat (Leica CM1860) and stored in a cryopreservant solution of PBS, ethylene glycol, and glycerol at −20°C. For immunofluorescence staining, brain sections were washed with PBS before incubation in a blocking solution of 10% donkey serum mixed with 0.5% Triton X-100. Overnight incubation at 4°C with primary antibodies followed this—anti-GABA (1:500, Sigma-Aldrich), anti-cFos (1:500, Synaptic Systems) in 3% donkey serum with Triton X-100. The sections were then incubated by the corresponding Alexa Fluor-conjugated secondary antibodies at room temperature for 1.5 h. For nuclear staining, slides were counterstained with DAPI (1:1,000, Sigma-Aldrich). After final washings, tissue sections were mounted and imaged on LSM880 and LSM980 confocal microscopes (ZEISS), with fluorescence signal quantification conducted using ImageJ (NIH). Fluorescence-positive cells were quantified by applying a threshold to grayscale images within 10% of the average intensity. Cells meeting or exceeding this threshold were considered positive.

### Statistical analysis

Statistical computations and graphics were produced using GraphPad Prism (version 8.0.2). The Shapiro–Wilk test was employed to evaluate the normality of data distribution. For normally distributed data sets, two-tailed unpaired Student’s *t*-tests were applied to compare the mean of two independent groups, or a two-way analysis of variance (Two-way ANOVA) followed by the Bonferroni *post-hoc* test to assess the significance of differences in the means of more than two independent groups. Data are presented as mean ± SEM, with significance thresholds set at *p < 0.05, **p < 0.01, and ***p < 0.001.

## Results

### Induction of tinnitus in mice by sodium salicylate injection

**Figure 1:**
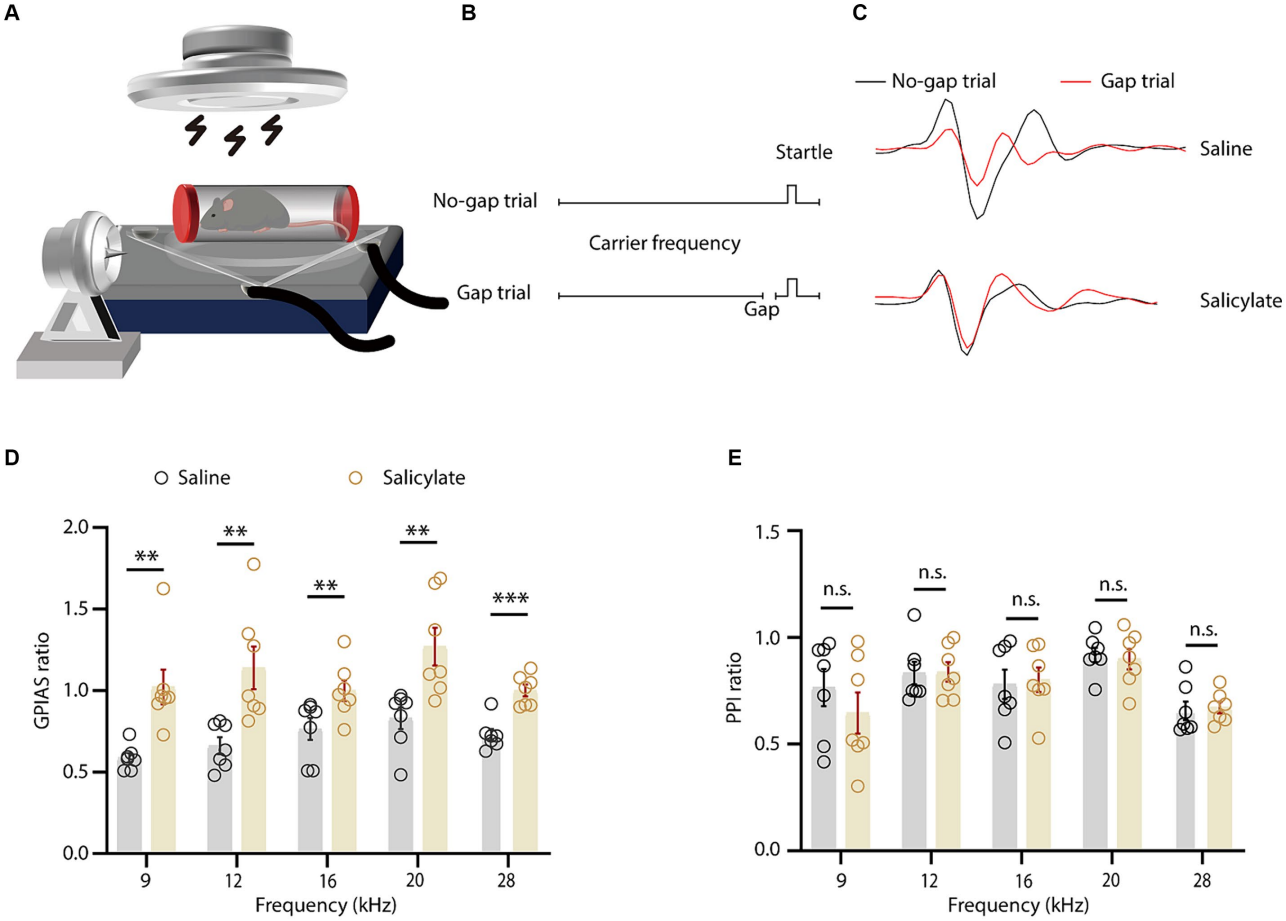
Behavioral assessment of salicylate-induced tinnitus in mice. **(A)** Schematic representation of the gap-prepulse inhibition of the acoustic startle (GPIAS) test. **(B)** The schematic showing the protocol for the GPIAS test. **(C)** The averaged voltage traces evoked by startle stimulus at different conditions as indicated. **(D)** The GPIAS ratio across various frequencies in mice administered saline or salicylate [treatment × frequency interaction, *F*_(4, 104)_ = 14.50, *p* < 0.0001; main effect of treatment, *F*_(1, 26)_ = 91.17, *p* < 0.0001, *n* = 7 mice/group]. **(E)** The pre-pulse inhibition (PPI) ratio at different background frequencies for saline or salicylate-treated mice [treatment × frequency interaction, *F*_(4, 104)_ = 0.1535, *p* = 0.9611; main effect of treatment, *F*_(1, 26)_ = 1.109, *p* = 0.3020, *n* = 7 mice/group]. Data are expressed as the means ± s.e.m. **p < 0.01; ***p < 0.001; n.s., not significant. Two-way ANOVA with Bonferroni *post hoc* analysis was used for **(D,E)**.

A single administration of sodium salicylate reliably induced tinnitus in mice, as evidenced by the altered startle reflex in a sound-proof chamber (Galazyuk and Hebert, 2015; Wang et al., 2019). The restraint apparatus, a custom-made tube with double rubber heads and lateral openings (Figure 1A), effectively transmitted sound stimuli to the mice. Gap Pre-pulse Inhibition of the Acoustic Startle (GPIAS) methodology indicated tinnitus-like behaviors in the experimental group. An increased startle inhibition ratio signaled the phantom perception of a sound akin to the background noise, indicative of tinnitus (Figures 1B,C). Compared to saline control, salicylate injection consistently elevated the startle ratio across all test frequencies, with statistically significant differences at frequencies of 9, 12, 16, and 28 kHz (Figure 1D). Additionally, the sensory gating function measured by pre-pulse inhibition (PPI) did not present significant differences in both salicylate treated and control groups after injection (Figure 1E).

### Mapping the auditory TRN pathways

**Figure 2:**
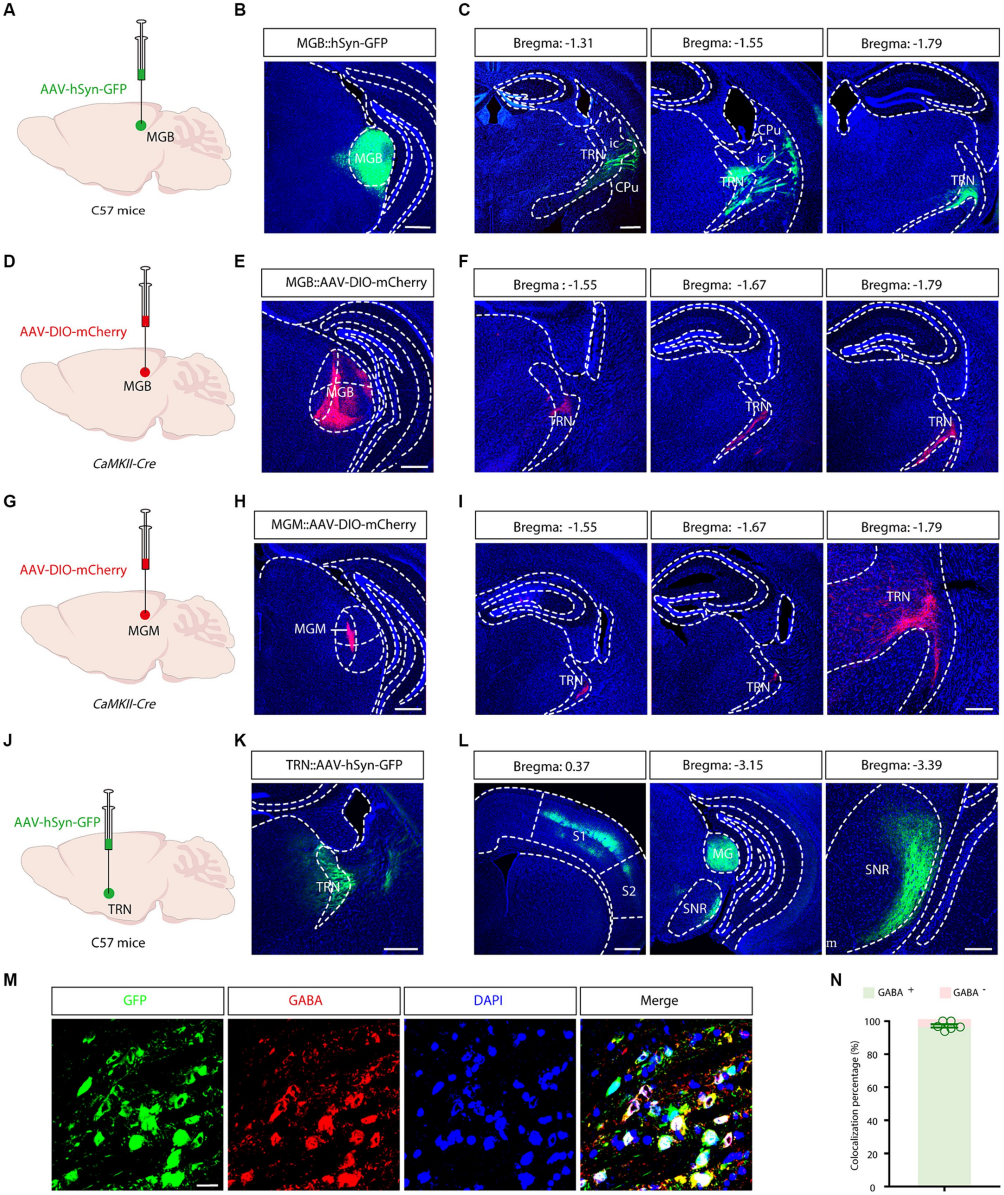
Morphological identification and output mapping of the auditory TRN. **(A)** Schematic illustrating viral injection into the medial geniculate body (MGB). **(B)** Microscopic image showing the site of viral injection in the MGB. Scale bar, 500 μm. **(C)** Projection fiber images in coronal sections containing the TRN at various bregma levels. Scale bar, 500 μm. **(D)** Schematic of viral injections into the MGB. **(E)** Image of the viral injection site. Scale bar, 500 μm. **(F)** Images displaying projection fibers in coronal sections through the TRN at different bregma locations. Scale bar, 500 μm. **(G)** Viral injection schematic for the MGM. **(H)** Image of the viral injection site. Scale bar: 500 μm. **(I)** Images displaying projection fibers in coronal sections through the TRN at different bregma locations. Scale bar: 500 μm (left), 200 μm (right). **(J)** Schematic illustrating viral injection into the auditory TRN. **(K)** Microscopic image showing the site of viral injection in the TRN. Scale bar, 500 μm. **(L)** Longitudinal images of projection fibers emanating from the TRN, Scale bar: 500 μm (left), 200 μm (right). **(M,N)** Typical images demonstrating co-localization of GABAergic neurons in the auditory TRN **(M)** and the summarized data **(N)**. Viral tracing was repeated in 3 mice. Scale bar: 20 μm. Cpu, caudate putamen; ic, internal capsule; MGB, medial geniculate body; MGM, MGB medial subdivisions; S1, primary somatosensory cortex; S2, secondary somatosensory cortex; SNR, substantia nigra; TRN, thalamic reticular nucleus.

The anatomical specificity of the aTRN was established through anterograde viral tracing. AAV-hSyn-GFP virus was introduced into the MGB of C57 mice (Figure 2A), resulting in proficient uptake and dense GFP-positive cell labeling in the MGB after 3 weeks (Figure 2B). GFP fibers were notably present in several brain regions, including the auditory cortex and the amygdala. TRN-specific MGB fibers were identified in coronal slices extending longitudinally from bregma −1.55 to −1.79 (Figure 2C). Consequently, the critical region of the TRN involved in conveying ascending auditory information, namely aTRN, was morphologically confirmed.

Next, separate output mappings were conducted in a cell-type and subdivision-specific manner. In *CaMKII-Cre* mice, after AAV-DIO-mCherry virus injection into the MGB (Figures 2D,E), mCherry-positive fibers were visualized in the TRN on coronal slices between bregma −1.55 and −1.79 (Figure 2F). A similar experiment performed in the medial subdivision of the MGB (MGM) revealed similar innervation patterns (Figures 2G-I). These results further confirmed the anatomical localization and the connectivity of the aTRN.

To delineate the cell types and outputs of the aTRN, C57 mice were subjected to AAV-hSyn-GFP injections. This anterograde tracing showed a high prevalence of GFP-positive neurons in the aTRN, confirming successful viral injections (Figures 2J,K). Further immunofluorescence staining established a predominant co-localization of aTRN neurons with GABAergic neurons (Figures 2M,N), validating that TRN is primarily inhibitory GABAergic neurons. GFP-positive fibers projecting to the primary somatosensory cortex (S1), the medial geniculate body (MGB), and the reticular part of the substantia nigra (SNr) were also observed (Figure 2L), indicating these downstream brain regions might be modulated by the auditory signals.

**Figure 3:**
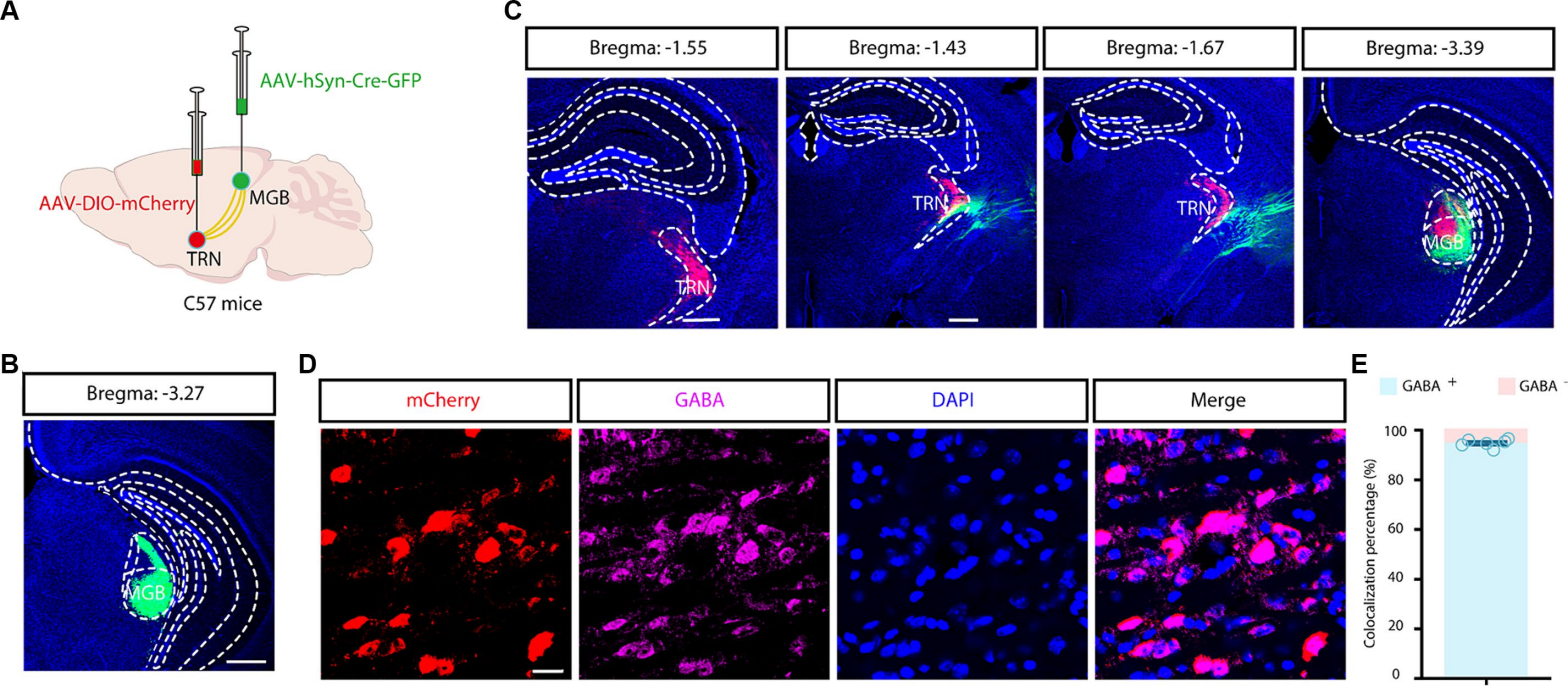
Combined viral strategy for tracing outputs from the auditory TRN. **(A)** Schematic depicting trans-synaptic anterograde tracing. **(B,C)** Fluorescence images of coronal brain sections along the MGB and TRN. Scale bar, 500 μm. **(D,E)** Images showing co-localization of auditory TRN neurons with GABA immunoreactivity **(D)** and the summarized data **(E)**. Viral tracing was repeated in 3 mice. Scale bar, 20 μm. MGB, medial geniculate body; TRN, thalamic reticular nucleus.

Nonetheless, the exclusivity of the aTRN was not guaranteed due to the potential non-specific spreading of the viruses. For precise targeting, a combined viral tracing strategy was performed in C57 mice by delivering a trans-synaptic anterograde AAV-hSyn-Cre-GFP virus into MGB and by delivering a Cre-dependent AAV-DIO-mCherry virus into the TRN (Figure 3A). Tracing results revealed widespread GFP-positive neurons in the MGB (Figure 3B) and substantial mCherry-positive neurons in the TRN (Figure 3C). These results confirmed that these mCherry-positive TRN neurons are directly downstream of the auditory thalamus. Further immunofluorescence established approximately complete co-localization of mCherry-positive neurons with GABAergic neurons (Figures 3D,E). Moreover, mCherry-positive fibers were evident in the MGB, reinforcing that MGB are key inhibitory targets for aTRN. This combined viral strategy allowed for the precise identification of aTRN neurons, ruling out the uncertainties associated with direct viral application in the presumptive aTRN region.

### Input mapping of auditory TRN

**Figure 4:**
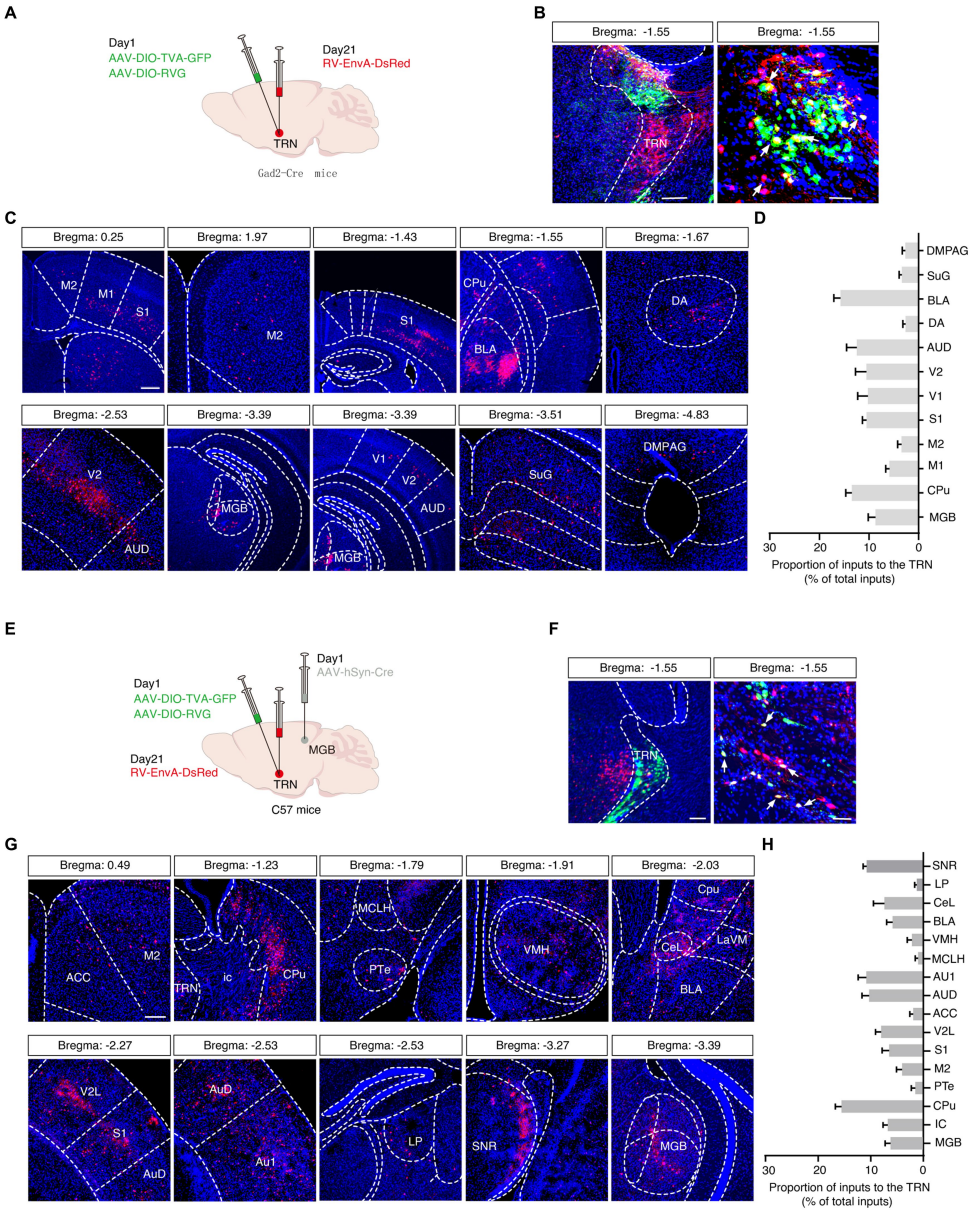
Viral tracing of inputs to the auditory TRN. **(A)** Schema of trans-synaptic rabies-based viral tracing. **(B)** Images of the starter cells at the TRN injection site (left, scale bar: 200 μm) and a magnified view (right, scale bar, 50 μm). **(C)** Visualization of DsRed-labeled signals along the rostral-caudal axis. Scale bar, 500 μm. **(D)** Summarized data for the proportion of virally traced neurons in different brain areas (*n* = 6 slices from 3 mice). **(E)** Schematic of combined trans-synaptic anterograde and rabies-based viral tracing. **(F)** Images of the starter cells at the TRN injection site (left) and a magnified view (right). Scale bar, 200 μm (left), 50 μm (right). **(G)** Images indicating DsRed signals traced in other brain regions longitudinally. **(H)** Summarized data for the proportion of virally traced neurons in different brain areas (*n* = 6 slices from 3 mice). ACC, anterior cingulate cortex; AUD, secondary auditory cortex, dorsal area; Au1, primary auditory cortex; BLA, basolateral amygdaloid nucleus CeL, central amydaloid nucleus, lateral part; Cpu, caudate putamen; DA, dorsal hypothalamic area; DMPAG, dorsomedial periaqueductal gray; ic, internal capsule; LaVM, lateral amygdaloid nucleus, ventromedial; LP, lateral posterior thalamic nucleus; M1, primary motor cortex; M2, secondary motor cortex; MCLH, magnocellular nucleus of the lateral hypothalamus; MGB, medial geniculate body; PTe, paraterete nucleus; S1, primary somatosensory cortex; SuG, superficial gray layer of the superior colliculus; SNR, substantia nigra; TRN, thalamic reticular nucleus; V1, primary visual cortex; V2, secondary visual cortex; V2L, secondary visual cortex, lateral area; VMH, ventromedial hypothalamus.

Next, we wanted to elucidate the inputs to the aTRN, and a rabies-based retrograde tracing method was utilized in *Gad2-Cre* mice in which Cre recombinase is expressed explicitly in GABAergic neurons (Figure 4A). Numerous GFP and DsRed co-expressing ‘starter’ cells were observed in the aTRN (Figure 4B), pointing to these cells being postsynaptic to traced DsRed neurons in other brain regions. DsRed-positive neurons were apparent in regions like the auditory cortex and MGB, confirming the auditory thalamocortical loop. Additionally, the presence of DsRed neurons in the amygdala, visual cortex, and somatosensory cortex suggested the possible influence of non-auditory areas on the aTRN (Figures 4C,D).

A second rabies-based strategy in C57 mice by delivering anterograde trans-synaptic AAV-hSyn-Cre virus into the MGB and by delivering helper viruses (AAV-EF1α-DIO-TVA-GFP and AAV-EF1α-DIO-RVG) into the aTRN was adopted to pinpoint the aTRN outputs further (Figures 4E,F). This approach not only confirmed the known brain regions projecting to the aTRN, such as the basolateral amygdala (BLA), auditory cortex (ACx), anterior cingulate cortex (ACC), central nucleus of the amygdala (CeA), substantia nigra (SNR), lateral posterior thalamic nucleus (LP), and MGB but also revealed numerous DsRed-labeled neurons in the ventromedial hypothalamus (VMH) and caudate putamen (Cpu) (Figures 4G,H), suggesting their involvement in auditory information processing.

### Chemogenetic modulation of salicylate-induced tinnitus via auditory TRN activation

**Figure 5:**
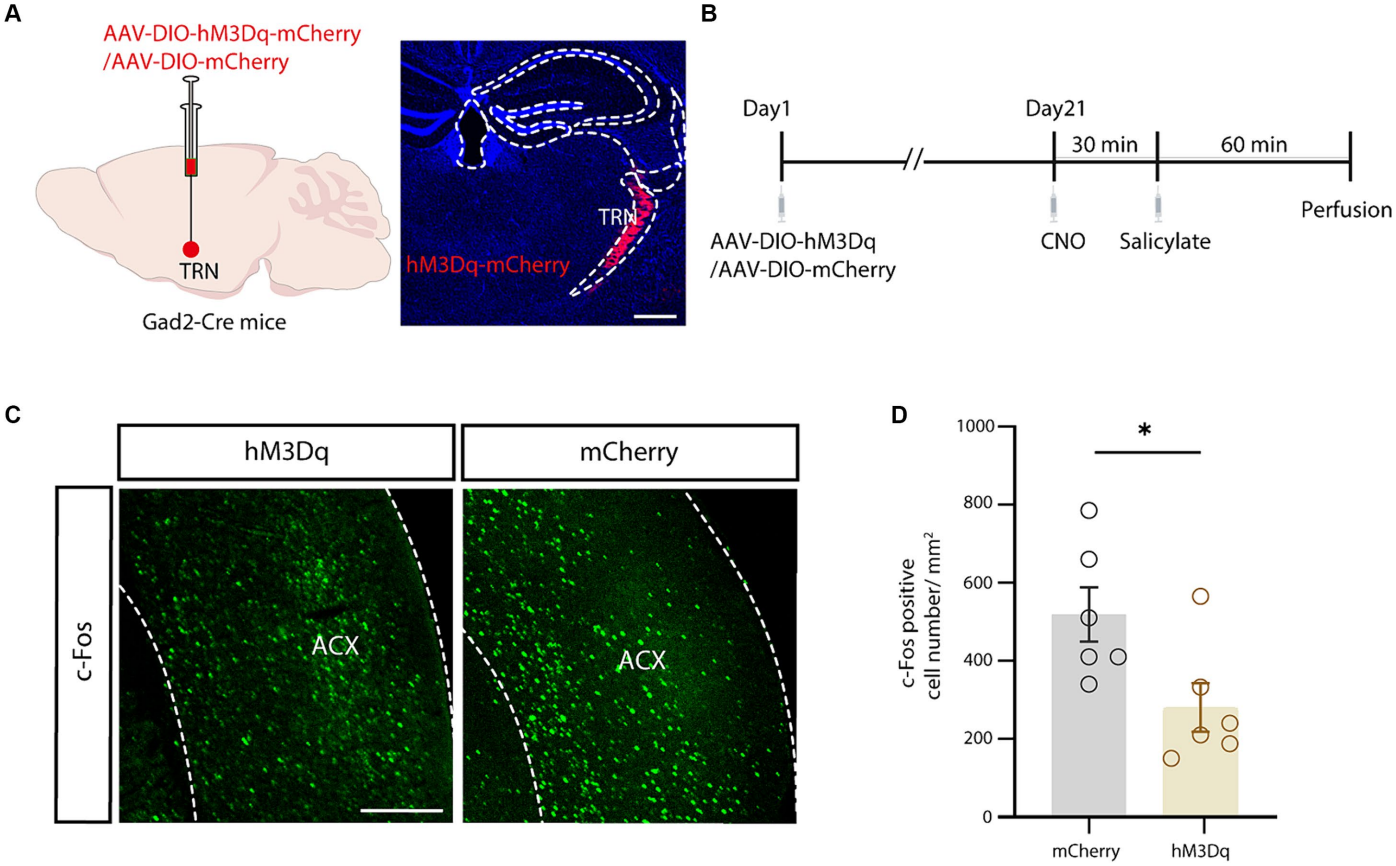
Chemogenetic activation of the auditory TRN mitigated salicylate-induced ACx neuronal hyperactivity. **(A)** Schematic for viral injection (left) and typical image of the viral injection region in the auditory TRN (right). Scale bar, 500 μm. **(B)** Schematic showing the experimental timeline. **(C)** Salicylate-induced c-Fos expression in the ACx in mice with auditory TRN expressing hM3Dq/mCherry (experimental and control mice) subjected to CNO injections. Scale bar, 500 μm. **(D)** Summarized cell counts of c-Fos positive cells in the ACx (*p* = 0.0290, *n* = 6 mice/group, unpaired Student’s *t*-test).

Finally, chemogenetics was applied to investigate the involvement of the TRN in salicylate-induced tinnitus. Cre-dependent excitatory hM3Dq virus was used to selectively excite the aTRN of *Gad2-Cre* mice (Figures 5A,B), in which systemic administration of sodium salicylate was used to induce tinnitus. The control mice with aTRN injection of AAV-DIO-mCherry received similar treatments of CNO and salicylate. Mice were sacrificed at 1h post-salicylate injection (Figure 5B), and the following immunofluorescence showed that chemogenetic activation of the aTRN substantially decreased the salicylate-evoked increase in c-Fos levels within the auditory cortex compared to control mice (Figures 5C,D). These findings suggest that artificial elevation of auditory thalamic inhibition could alleviate physiological alterations associated with salicylate-induced tinnitus.

## Discussion

This study provides a morphological characterization of the auditory thalamic reticular nucleus (TRN) and examines the modulation of salicylate-induced tinnitus via TRN-mediated inhibition. Employing advanced anterograde and retrograde viral tracing techniques, we delineated the anatomic localization of the auditory TRN and mapped its synaptic input and output networks. The precise boundaries and connections of the auditory TRN were elucidated using a strategic trans-synaptic anterograde spread of Cre recombinase from the medial geniculate body (MGB) to the TRN and subsequent Cre-dependent virus expression. This methodology, complemented with rabies-based retrograde tracing, accurately traced the brain regions innervating the auditory TRN. Furthermore, chemogenetic excitation of the TRN significantly mitigated the enhanced neural activity in the auditory cortex caused by salicylate, demonstrating the potential of enhancing TRN-mediated inhibition to alleviate tinnitus.

In the current study, we majorly examined tinnitus in mice receiving systemic salicylate injection. Salicylate-induced tinnitus is known for its reversibility, subsiding after salicylate withdrawal (Stolzberg et al., 2012). Additionally, salicylate overdose is now uncommon. On the other hand, tinnitus related to hearing loss caused by noise exposure represents a more realistic situation (Eggermont, 2008). Although salicylate-induced tinnitus may not fully capture the complexity of human tinnitus, it does share common mechanisms with tinnitus of other etiologies, such as increased spontaneous firing rates in auditory neurons and changes in tinnitus-associated brain regions (Stolzberg et al., 2012). Therefore, studying salicylate-induced tinnitus provides valuable insights into the neural mechanisms underlying tinnitus, contributing to our understanding of the condition across different etiologies. Both single (Yang et al., 2007; Lu et al., 2011; Su et al., 2012; Sun et al., 2014; Liu and Chen, 2015) and multiple (Yi et al., 2016) doses of salicylate have been successfully used to induce tinnitus-like behaviors, which necessitate the use of a reliable, objective measure for tinnitus (Jastreboff et al., 1988). The GPIAS has been developed based on the acoustic startle response (Galazyuk and Hebert, 2015). It has been observed that the acoustic reflex is diminished when it is preceded by a silent gap embedded in background noise. Tinnitus, similar in quality to the background noise, “fills in” the gap and reduces inhibition. However, it is essential to note that GPIAS relies considerably on the individual’s hearing level, and caution should be exercised when studying subjects with hearing loss (Galazyuk and Hebert, 2015; Rogenmoser et al., 2022). Various behavioral training methods such as conditioned level or lick suppression (Ruel et al., 2008; Wang et al., 2019), place preference (Yang et al., 2011), two-alternative choice (Stolzberg et al., 2013), and tactile reflex (Rogenmoser et al., 2022) have been utilized to screen tinnitus-like behaviors accurately.

The auditory thalamus gates auditory information, reaching the cortex (He, 2001). The TRN, composed entirely of inhibitory neurons, modulates the thalamocortical and corticothalamic information flow (Steriade et al., 1993; Llinas et al., 2005). The interconnected network of the TRN, dorsal thalamus, and cortex is pivotal in generating neural oscillations (Steriade et al., 1993; Guo et al., 2007). Disruption in this thalamocortical circuitry is linked to altered neural synchronization, evident in pathological conditions such as tinnitus, where aberrant gamma oscillations may manifest due to functional changes within this network (Llinas et al., 2005; Weisz et al., 2007; Roberts et al., 2010; Vianney-Rodrigues et al., 2019; Hayes et al., 2021).

Traditionally, the auditory TRN has been approximately identified via conventional tracing techniques or electrophysiological response recordings (Yu et al., 2009a; Kimura et al., 2012). However, we can now map the auditory TRN and its networks with modern viral vectors with greater precision. Our trans-synaptic virus injections at the auditory thalamus allowed a precise definition of the auditory TRN, and Cre-dependent mapping strategies further specified its input and output channels (Xu et al., 2020; Liu et al., 2022). This combined tracing strategy delineated projections accurately, in contrast to the potential confounds of direct TRN viral injections. Our findings reveal that the auditory TRN projects inhibitory signals predominantly to the MGB while maintaining connections with a variety of brain regions, including the central and basolateral amygdala, cingulate cortex, dorsal hypothalamic area, visual cortex, and somatosensory cortex. The specific roles these connections play in auditory function merit further exploration.

Like phantom limb pain (De Ridder et al., 2011), hearing loss-induced tinnitus is marked by cortical reorganization and altered inhibition (Rajan, 1998; Eggermont and Roberts, 2004; Weisz et al., 2007; Rauschecker et al., 2010; Roberts et al., 2010). Sodium salicylate-induced tinnitus, particularly, is tied to diminished auditory inhibition (Wang et al., 2006, 2008; Sun et al., 2009; Asim et al., 2023). Past studies have highlighted how noise exposure and aging can diminish auditory system inhibition (Middleton et al., 2011; Llano et al., 2012; Asim et al., 2023), with salicylate directly attenuating inhibitory synaptic transmission in the auditory cortex and inferior colliculus—implicating weakened inhibition as a mechanism in tinnitus (Wang et al., 2006, 2008, 2011). Given the pivotal role of TRN inhibition in auditory processing and its implications in the imbalance between excitation and inhibition in tinnitus, strategic modulation of this central inhibition could provide relief (Rauschecker et al., 2010; Yang et al., 2011; Minen et al., 2014). This notion is supported by the efficacy of treatments like Vigabatrin, which enhances GABA receptor function (Brozoski et al., 2007), aligning with our observations that chemogenetic excitation of the TRN softens salicylate-induced cellular hyperactivity. However, our study does have several limitations that should be acknowledged. Firstly, salicylate also induces hearing loss, which may compromise the verification of tinnitus-like behavior through the GPIAS protocol that relies on auditory perception. Therefore, there is a need to develop more reliable behavioral protocols for validating salicylate-induced tinnitus behaviors (Galazyuk and Hebert, 2015; Rogenmoser et al., 2022). Secondly, we only verified salicylate-induced tinnitus-like behavior in wild-type mice and did not assess this in Gad2-Cre mice, in which the modulatory effects of aTRN on tinnitus were examined in the current study (Masri et al., 2021). Thirdly, CaMKII-Cre mice were used to transfect the excitatory neurons in the MGB, but the more suitable transgenic mice should be Vglu2-Cre mice, which are uniquely expressed in glutamatergic neurons of the central nervous system. Lastly, while the AAV-hSyn-Cre-GFP virus is known to be transported anterogradely (Zingg et al., 2017, 2020), it would also be beneficial to investigate its reported retrograde properties in future studies.

Our study maps the auditory TRN’s anatomical localization, inputs, and outputs with unprecedented precision. The modulation of TRN activity shows promise in counteracting the neural hyperactivity associated with salicylate-induced tinnitus, suggesting that the auditory TRN pathway could be a potential therapeutic target for this condition. A deeper understanding of the aTRN’s neural networks may lead to developing a practical innervation approach to enhance TRN inhibition, offering hope for individuals affected by tinnitus.

## Data availability statement

The original contributions presented in the study are included in the article/supplementary material, further inquiries can be directed to the corresponding authors.

## Ethics statement

The animal study was approved by the Animal Care Committee of Anhui University of Chinese Medicine. The study was conducted in accordance with the local legislation and institutional requirements.

## Author contributions

QD: Writing – original draft, Writing – review & editing. TQ: Writing – original draft, Writing – review & editing. GS: Writing – original draft, Writing – review & editing. HW: Conceptualization, Writing – original draft, Writing – review & editing.
